# Trends in antibiotic susceptibility patterns and epidemiology of MRSA isolates from several hospitals in Riyadh, Saudi Arabia

**DOI:** 10.1186/1476-0711-5-30

**Published:** 2006-12-02

**Authors:** Manal M Baddour, Manal M Abuelkheir, Amal J Fatani

**Affiliations:** 1 Microbiology and Immunology Dept, Faculty of Medicine, Alexandria University, Egypt; 2 Microbiology Dept, King Saud University, Women Student's Medical Studies and Sciences Sections, Riyadh 11495, P.O. Box 11495, Saudi Arabia; 3 Pharmacology Dept, King Saud University, Women Student's Medical Studies and Sciences Sections Riyadh11495, P.O. Box 11495, Saudi Arabia

## Abstract

**Background:**

Methicillin-resistant *Staphylococcus aureus *(MRSA), is associated with high morbidity and mortality rates with rapid development of resistance.

**Methods:**

A total of 512 MRSA isolates were procured from 6 major hospitals in Riyadh, Saudi Arabia and antibiotic susceptibilities and MICs were documented against several antibiotics and vancomycin. SPSS version 10 was used for statistical analysis.

**Results:**

The prevalence of MRSA in the study hospitals ranged from 12% to 49.4%. Mean patient age was 44 years with males constituting 64.4% and females 35.6%. Approximately 41.5% of the isolates came from patients in the extreme age groups. MIC for vancomycin was in the susceptible range for all isolates ranging from 0.25 to 3 ug/ml. The overall susceptibility of MRSA to the various antibiotics tested was: fusidic acid 4.3%, sulfamethoxazole/trimethoprim 33.8%, gentamicin 39.6%, mupirocin 77.0%, gatifloxacin 78.9%, chloramphenicl 80.7%, linezolid 95.1%, quinupristin/dalfopristin 100%. Some differences were noted in the resistance of isolates among the participating hospitals reflecting antibiotic usage. On the whole, inpatient isolates (accounting for 77.5% of the isolates) were more resistant than outpatient isolates (22.5%) except for linezolid. Quinupristin-dalfopristin and linezolid are the most effective antibiotics tested against inpatient isolates while quinupristin-dalfopristin and gatifloxacin seem to be the most effective against outpatient isolates.

Approximately one forth of the isolates are no longer susceptible to mupirocin used for eradication of the carrier state reflecting resistance developing after widespread use. Trends over time show a tendency towards decreased susceptibility to gatifloxacin and linezolid with increasing susceptibility to gentamicin and sulfamethoxazole/trimethoprim.

**Conclusion:**

Quinupristin/dalfopristin and linezolid are two valuable additions to our antimicrobial armamentarium, but resistance has already been described. To preserve their value, their use should be limited to those rare cases where they are clearly needed. Fusidic acid, the local antibiotic, gentamicin and trimethoprim/sulfamethoxazole should not be relied upon for treatment of MRSA infections, at least empirically as the percentage of susceptible isolates is very low.

## Background

*Staphylococcus aureus *(*S. aureus*) is a major pathogen associated with serious community- and hospital-acquired diseases. Most of *S. aureus *infections are caused by methicillin sensitive *Staphylococcus aureus *strains (MSSA) that are susceptible to all other classes of anti-staphylococcal antibiotics. Methicillin resistant *Staphylococcus aureus *strains (MRSA) are implicated in serious infections and nosocomial outbreaks. These strains show resistance to a wide range of antibiotics, thus limiting the treatment options to very few agents such as vancomycin and teicoplanin[1,2].

Microbes have genetic plasticity, which means that they have the capacity to evolve in response to their environment. The major impetus for developing resistance is selective pressure resulting from antibiotic use. The bacteria that survive are those that develop mechanisms to avoid being killed by antibiotics. The treatment of several pathogens, including MRSA, is problematic. New solutions are needed to preserve the activity of our current antibiotic armamentarium, to lower the overall risk of bacterial resistance and to successfully treat patients with resistant bacterial infections. Options include: development of new antibiotics to treat resistant organisms; vaccination to prevent infections; and improved use of antibiotics. Because bacteria will eventually develop means to avoid being killed by antibiotics, judicious use of antibiotics by all clinicians is imperative. Appropriate antibiotic use involves selection of a "targeted spectrum" antibiotic, as well as an appropriate dose and duration. This entails updated databases on the antibiotic susceptibility of such databases to new as well as traditional antibiotics[3].

Because the mechanism of resistance is an alteration in the target of the antibiotic, MRSA are resistant clinically to all beta-lactam antibiotics, even though a drug such as cefazolin may appear to be active in vitro. It is also important to note that MRSA are often multidrug-resistant and are resistant to antibiotics such as the macrolides and aminoglycosides, even though the mechanisms of action of these antibiotics are different than that of the beta lactams.

Clinical isolates of MRSA that are intermediate to vancomycin, called vancomycin-intermediate *Staphylococcus aureus *(VISA), were first identified in patients in Japan in 1996[4]. As of June 2002, 8 VISA infections had been documented in patients in the US[5]. Vancomycin has a narrow spectrum of activity, restricted to most Gram-positive bacteria, and is the drug of choice for the treatment of (MRSA). The vancomycin MIC for MRSA is 1–2 mg/L for fully vancomycin-susceptible strains. Vancomycin inhibits peptidoglycan synthesis by binding to the D-Ala-D-Ala terminus of the nascent murein monomer, resulting in the inhibition of cell-wall synthesis. Only 50% of the vancomycin arriving at the surface of a staphylococcus will reach the target site. VISA are characterized by a thicker cell-wall with increased amounts of peptidoglycan, and the increased quantities of unprocessed D-Ala-D-Ala cause increased 'trapping' and 'clogging', resulting in higher vancomycin MICs of 8–16 μg/ml and the increased inoculum effect observed with VISA in comparison with fully vancomycin-susceptible strains[6].

In June 2002 the first clinical isolate of vancomycin resistant *Staphylococcus aureus *(VRSA) was reported from a patient in Michigan[5]. The term VRSA is based on the vancomycin breakpoint of the British Society for Chemotherapy, where a strain for which the MIC is 8 mg/liter is defined as resistant. Since the same MIC is defined as indicating intermediate susceptibility by the NCCLS, these VRSA strains are called vancomycin-intermediate *Staphylococcus aureus *or glycopeptide-intermediate *Staphylococcus aureus *in the United States[7].

Early observations from both clinical isolates and laboratory-derived strains of GISA have focused on the bacterial cell wall, where the glycopeptides exert their antimicrobial effect. The glycopeptides prevent the transglycosylation and transpeptidation reactions necessary for the formation of mature cell wall in Gram positive bacteria. Specifically, they bind to the D-alanyl-D-alanine terminus of the N-acetylmuramyl pentapeptide subunit of the nascent cell wall. On the basis of these and other observations, Sieradzki *et al*. (1999)[8], proposed a functional model in which glycopeptide molecules are first "captured" in the cell wall, then serve to block access of other glycopeptide molecules to nascent cell wall elements. Additional investigation of laboratory derived vancomycin-resistant strains demonstrated down-regulation of certain penicillin-binding proteins, including PBP2A.

Quinupristin/dalfopristin (Synercid) is a semisynthetic antibiotic that combines two streptogramin compounds in a 30:70 ratio, quinupristin (a group B streptogramin) and dalfopristin (a group A streptogramin), and is the first licensed antibiotic in its class. It inhibits bacterial protein synthesis by binding of each component to a different site on the 50S subunit of the bacterial ribosome, dalfopristin leading to a conformational change in the ribosome which increases the affinity of the ribosome for quinupristin. Each of the two streptogramins separately acts as a bacteriostatic agent but in combination they are bactericidal.

Quinupristin/dalfopristin is available only as an intravenous product. Its spectrum of activity is similar to that of vancomycin, with excellent activity against Gram positive pathogens, including many resistant strains, such as MRSA[9]. Its major value is that it provides a therapeutic option for infections caused by vancomycin-resistant *Enterococcus faecium*, VISA or VRSA. Unfortunately there are already reports of VRE and MRSA resistant to quinupristin/dalfopristin since its licensure in 1999[10,11].

Linezolid (Zyvox) is the first licensed oxazolidinone antibiotic. The oxazolidinones, synthetic compounds unrelated to other antimicrobials, inhibit bacterial protein synthesis by binding to the ribosome 50S subunit, thus blocking the initiation complex formation. Linezolid has limited activity against selected Gram-negatives and anaerobes but is highly active against Gram-positive bacteria, including resistant strains. Like quinupristin/dalfopristin, linezolid is active against MRSA, but is only bacteriostatic. Linezolid is available in both intravenous and oral preparations and is 100% bioavailable after oral administration. As such it provides an oral therapeutic option for patients with Gram-positive infections resistant to other oral antibiotics. Linezolid lacks cross-resistance to any other group of antibiotics. Since linezolid became available in 2000, clinical isolates of VRE and MRSA resistant to linezolid have been reported from treated patients [12-14].

Although the fluoroquinolones are not new antibiotics, many studies are still being conducted to assess their uses. Important features of this drug class include excellent bioavailability after oral administration, achievement of high tissue concentrations and a broad spectrum of activity. In general fluoroquinolones are active against many Gram-positive bacteria. They do not appear to be affected by β-lactamase enzymes or altered penicillin binding proteins. The quinolones have a unique mechanism of action; they inhibit two bacterial enzymes, DNA gyrase and topoisomerase IV, that are essential for bacterial DNA synthesis. Because they target bacterial sites distinct from the site of action of other antibiotics, it was hypothesized by some that resistance might be less likely to occur or slower to develop[15]. Unfortunately these hopes were not borne out.

Mupirocin is a naturally occurring agent produced by *Pseudomonas fluorescens *and has successfully been used to reduce substantially the nasal and hand carriage of MRSA[16,17]. This regimen is least effective in patients with either indwelling catheters or lesions on their skin. Mupirocin (pseudomonic acid) specifically binds to bacterial isoleucyl-tRNA synthetase (IRS) and inhibits protein synthesis[18]. However, emergence of mupirocin-resistant MRSA strains as a result of long-term and intermittent usage of the antibiotic has also been reported[19,20]. Repeated courses of topical antimicrobial treatment should be discouraged as they often lead to emergence of strains of bacteria that are resistant to these agents[21]. However, Fawley et al[22], 2006 provide evidence that short-term mupirocin prophylaxis may be helpful in the prevention of *S. aureus *surgical site infections with little chance of risk of resistance selection.

Extensive anecdotal data support the use of trimethoprim/sulfamethoxazole for infections caused by MRSA, but only one randomized clinical trial has demonstrated its efficacy for such infections[23].

A detailed knowledge of the susceptibility to antimicrobial agents is necessary to facilitate the development of effective strategies to combat the growing problem of resistance. A nationwide knowledge base is also important for optimal patient management, control of nosocomial infection and for the conservation of antibiotics. This study was thus designed to track the resistance trends of MRSA isolates from different hospitals to the non-beta-lactams that are commonly used to combat infections by it.

## Methods

Five hundred and twelve MRSA isolates were consecutively procured from samples submitted to the microbiology labs from patients being treated in several tertiary care hospitals with different geographical locations within Riyadh. The hospitals were designated the code names Hospitals A to F. The names of the hospitals were not stated for privacy reasons and are available from the authors upon request. Isolates were collected during the period from January 2004 through December 2005. No duplicate isolates from the same patient and no environmental strains were included in this study. The methicillin resistant *S. aureus *ATCC 33591 was included as a reference strain for quality control. Isolates were identified as *S. aureus *by the standard microbiological procedures[24]. Then the following tests were carried out:

### I- Detection of methicillin resistance

This was carried out according to NCCLS guidelines using Oxacillin agar screen test whereby all MRSA isolates were spot inoculated onto Mueller-Hinton agar supplemented with 6 μg/ml oxacillin and 4% NaCl, from a 0.5 McFarland standard suspension. The plates were incubated at 35°C for 24 h as recommended by the Clinical Laboratory Standards Institute (CLSI), formerly NCCLS. If any growth (more than one colony) was detected, the isolate was considered oxacillin or methicillin resistant[25].

### II- Surveillance of MRSA with decreased vancomycin susceptibility

Vancomycin resistance was tested for by vancomycin agar screening test whereby MRSA isolates were spot inoculated onto Mueller Hinton agar supplemented with 6 μg/ml of vancomycin from a 0.5 McFarland standard suspension. The plates were incubated at 35°C for 24 h as recommended by the NCCLS. Any isolates growing two or more colonies on this agar would be considered as positive[25].

### III- Evaluation of Antibiotic susceptibility patterns

Various antibiotics including traditional as well as recently introduced ones were used in disc diffusion tests (Oxoid) according to NCCLS guidelines against all isolates to determine the susceptibility of these isolates to such antibiotics[25].

The antibiotics tested included: gatifloxacin, gentamicin, linezolid, quinupristin-dalfopristin, mupirocin, fusidic acid, chloramphenicol and trimethoprim-sulfamethoxazole.

### IV- MIC determination

Determination of the MIC against vancomycin to detect any isolate with a decreased susceptibility to the drug using E-test (AB-Biodisk, Solna, Sweden). The tests were performed according to the manufacturer's instructions. E-test for the other tested antimicrobials except fusidic acid and chloramphenicol as well as E-test for minocycline were performed for select susceptible strains of MRSA to give an idea about the MIC in our tested isolates.

### Statistical methods

Statistical package for social sciences (SPSS) version 10 was used to analyze our data. Comparison of categorical variables and percentages between groups was done by the Pearson chi-square test or Fisher's exact test, as appropriate. Logistic regression analysis was carried out to find association between variables. The threshold for a significant difference was designated a *P *value of <0.005. All tests were two tailed.

## Results and Discussion

MRSA isolates from inpatients accounted for 77.5% of the isolates (397/512), while 22.5% came from outpatients (115/512). Inpatient isolates were distributed in the following services: ICU: 96 (24.2%), Medicine: 59 (14.9%), Surgery: 54 (13.6%), Pediatric: 48 (12.1%), Burn & Plastic Surgery: 29 (7.3%), Orthopedic Surgery: 27 (6.8%), Renal: 18 (4.5%) & other unspecified wards: 66 (16.6%). Most isolates came from wounds (39.7%) followed by soft tissues (28.4%).

Regarding the gender distribution of the isolates, 64.4% were recovered from male patients while 35.6% were from females. These values are quite similar to those reported by van Belkum et al[26], 1997 from King Faisal Specialist hospital – which was one of the hospitals included in the present study – isolated from patients referred to it from several other hospitals in Saudi Arabia. They report procurement of 66% of their isolates from male patients and 34% from females. Madani et al[27], 2001 also report a 65.8% recovery from males and 34.2% from females in Saudi Arabia. Similarly, from the eastern province of Saudi Arabia, Bukharie & Abdelhadi[28] (2001) report 63% of MRSA isolation from males and 37% from females so this probably reflects the distribution of MRSA throughout the Kingdom with a male patient predominance most likely due to the fact that exposure is greater. This gender distribution was also similar to that reported by Tentolouris et al[29], 2006 where 60.7% were males and 39.3% were females.

The mean age of the study group was 44 years with an age span from <1 to 95 years old. This is higher than the mean age reported by Bukharie & Abdelhadi (35.7y)[28]. Approximately 41.5% of the isolates came from patients in the extreme age groups, 21.0% ≥ 60 years and 20.5% ≤ 5 years. Madani et al[27], 2001 similarly report isolation of 26.1% of MRSA from patients ≥ 60 years and 26.1% from patients ≤ 1 year in another Saudi population. This has likewise been reported by Kuehnert et al[30], 2005 from the USA whereby most MRSA diagnosis occurred in persons ≥ 65 years of age. Discordantly, Tentolouris et al[29], 2006 report a much higher mean age of 60.1 years.

The prevalence of MRSA among *S. aureus *isolates varied from one hospital to another and ranged from 12% to 49.4% with 4 hospitals lying in the range of 27–33%. Hospital A was the hospital from which the highest prevalence was encountered and this is expected due to the fact of it being a referral hospital for most other Ministry of Health hospitals within and around Riyadh. The 27–33% range is quite similar to the 33% reported earlier from Jeddah, Saudi Arabia in 2001[27] and 2003[31], as well as 31% in 2005[32]. Yet others report the much lower prevalence of 12% in 2001 from the eastern province[28]. The same prevalence is reported from Nigeria, Kenya and Cameroon[33]. MRSA prevalence is generally reported to be high in North America (43.7% & 43.2%)[30,34], southern European countries[35,36], Japan (50–70%)[37], Malaysia[38], Latin America[39], Ethiopia[40], Sri Lanka[41]. In fact, according to the National Nosocomial Infection Surveillance System (NNIS) report, 50% of hospital acquired infections in ICUs in the USA are due to MRSA[42]. In other countries such as Tunisia, Malta, Algeria[33], Sweden, Switzerland, the Netherlands (the SENTRY participants group, 2001)[43] and Australia (14.9%)[44] on the other hand, it is low. In developing countries, it has always been contended that the inappropriate use of antibiotics for community infections may further increase the pressure to select MRSA and other resistant bacteria. Yet the higher prevalence of MRSA reported from other more developed countries argues against this and perhaps points out to the fact that injudicious use of antibiotics stands true not only for community infections but is true for prescription as well as over the counter medicines. Bacterial resistance threatens our ability to treat both common and serious infections. Although new antibiotics can effectively treat some resistant pathogens and more research is needed to develop novel antimicrobials, bacteria will eventually develop resistance to any antibiotic with time. The misuse and overuse of antibiotics drive the emergence and spread of resistance. Eliminating inappropriate antibiotic use and promoting more judicious use are essential parts of the solution.

For all the acquired isolates, screening for oxacillin resistance has been re-documented using the oxacillin agar screening test using a Mueller-Hinton medium with 4% NaCl and 6 μg/ml oxacillin according to NCCLS guidelines.

Similarly, screening for vancomycin resistance has been carried out using Mueller Hinton agar plates plus 6 μg/ml vancomycin. Until now, no such isolates have been detected nor have they been reported by other researchers in Saudi Arabian hospitals[28]. This is reassuring and indicates that VRSA has not yet set foot in the Saudi hospitals studied unlike reports from Japan[45], United States[4], Europe and the Far East[46]. Results of the vancomycin E-test showed that all isolates were susceptible with MICs ranging from 0.25 μg/mL to 3 μg/mL, the higher MICs mainly being from Hospital A.

Determining the in vitro activity of new antimicrobial agents against pathogens showing increasing resistance to other compounds is important when the global escalation of this trend is considered. Hence the CLSI M39-A guidelines recommend that antibiogram data should be analyzed at least annually, thus determination of the antibiotic susceptibility patterns of the procured isolates against some non-β lactams was performed according to the NCCLS guidelines and results of the susceptibility testing are shown in table 1.

**Table 1:** Antibiotic susceptibility results of the tested isolates

**Antibiotic**	**Total susceptibility No. (%) (512)**	**Inpatient isolates No. (%) (397)**	**Outpatient isolates No. (%) (115)**
Vancomycin	512(100)	397(100)	115(100)
Quinupristin/dalfopristin	512(100)	397(100)	115(100)
Linezolid	491(95.9)	386(97.2)	105(91.3)
Chloramphenicol	413(80.7)	302(76.1)	111(96.5)
Gatifloxacin	404(78.9)	299(75.3)	105(91.3)
Mupirocin	394(77.0)	292(73.6)	102(88.7)
Gentamicin	203(39.6)	118(29.7)	85(73.9)
Sulfamethoxazole/trimethoprim	173(33.8)	85(21.4)	88(76.5)
Fusidic acid	22(4.3)	15(3.8)	7(6.1)

**Figure 1:**
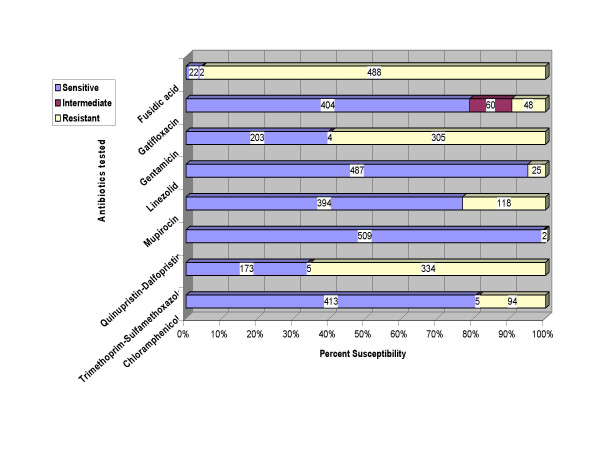
Comparative susceptibility of the 512 MRSA isolates to tested antimicrobials.

As depicted in table 1 and figure 1, 78.9% of the isolates were susceptible to gatifloxacin (isolates with intermediate resistance were included with the resistant ones). This is in contrast to the high resistance rates of MRSA isolates from Japan to fluoroquinolones which are at the high 80–95%[47], which probably reflects the excessive use of this class of antibiotics there and thus induction of resistance. In North America, gatifloxacin susceptibility is 64.7%[34], which is closer to our results. Susceptibility to chloramphenical in the Japanese isolates ranged from 3.8% to 5.1%[47], while in the present study, 80.7% of MRSA were susceptible. Panhotra et al, from Al-Hasa region of Saudi Arabia report full susceptibility of their MRSA isolates to chloramphenicol[48]. Linezolid was highly effective in the present study with an overall 95.9% susceptibility and was also reported in 2005 from Poland and in 2006 from UK to be fully susceptible[49,50]. Isolates showed a 77.0% susceptibility to mupirocin, this is in between the 83.4% reported from Austria, Germany and Switzerland[51], the 88.9% reported from the UK[22] and the 71.9% reported from Kuwait[52]. Gentamicin was poorly effective against our MRSA isolates (39.6%) and gave even weaker results reported in 2001 (34.8%)[28], and 2005 (0% & 25%)[48,49]. Results given by trimethoprim-sulfamethoxazole are even worse with a mere 33.8% susceptibility in the current study, 21.1% from Bukharie and Abdelhadi[28], 2001 and full resistance by Panhotra et al, 2005[48]. Our results are in sharp contrast with those of Echa'niz-Aviles et al[53], 2006 who found all their isolates to be susceptible to gentamicin and trimethoprim-sulphamethoxazole. It is pertinent to deduce that antibiotics such as gentamicin and trimethoprim-sulfamethoxazole and the local fusidic acid should no longer be relied upon at least for empirical treatment of the local MRSA isolates. Whether the resistance observed in tested isolates comes from their inherent genetic propensity to acquire resistance or this is due to mere selection of antibiotic resistant isolates through monotherapy or under-dosage could not be clarified as the previous antibiotic intake data were not available for all isolates.

Table 1 also shows the percent susceptibilities of the MRSA isolates from inpatients versus isolates from outpatients. Susceptibilities of MRSA against all antibiotics tested was higher for outpatient as opposed to inpatient isolates except for linezolid. This was profoundly evident for gentamicin and trimethoprim/sulfamethoxazole. It was also evident for mupirocin, the local antibiotic used for eradication of the carrier state which is expected due to its use in the hospitals. This was also evident for gatifloxacin, the fluoroquinolone, and again use of fluoroquinolones and thus appearance of resistance against them is expected in hospital isolates. It has been reported in the The Medical Letter On Drugs and Therapeutics that in adequate dosage, sulfamethoxazole/trimethoprim appears to be effective against CA-MRSA, and that resistance is rare, this was the case in the present study where 77.4% of the outpatient isolates were susceptible to sulfamethoxazole/trimethoprim while only 22.2% of the inpatient isolates were susceptible to it.

While the collection of MRSA did not specifically determine community versus nosocomial isolates, it could be generally expected that most outpatient isolates would be community acquired while most inpatient isolates would be nosocomial and thus we can deduce that hospital isolates are more resistant than community isolates.

Table 2 shows the percentage susceptibilities of the isolates from the different hospitals included in the study to the antibiotics tested by the disc diffusion method. From the table, wide variations are observed between the hospitals regarding susceptibility to some antibiotics such as gatifloxacin which was apparently effective for most hospital isolates except for Hospital E where only 52.7% of the isolates were susceptible and Hospital D where only 56.3 % of the isolates were susceptible. This difference was statistically significant (*p *< 0.005). This seems to reflect a high usage of fluoroquinolones in these hospitals. For quinupristin/dalfopristin, all of the hospital isolates were 100% susceptible. The level of mupirocin susceptibility was in the range of 71 to 88% for most of the hospitals, which probably also reflects high usage as an infection control measure. It has been shown previously that in hospitals where mupirocin use is common the percentage of mupirocin resistant isolates can be extremely high (63%)[54]. Surprisingly though, Caierao et al[55], 2006 report an actual decrease in the level of mupirocin resistance during regular use in ICU. Wide variations in the susceptibility of isolates to sulfamethoxazole/trimethoprim and gentamicin were observed among hospitals, while susceptibility to chloramphenicol and linezolid as well as resistance to fusidic acid were fairly similar.

**Table 2:** Percentage susceptibility of MRSA isolates from the studied hospitals to the antibiotics tested by disc diffusion according to CLSI standards

Hospital code	FD	GAT	GEN	LZD	MUP	Q/D	SXT	CHL
A (179)	3.4	84.9	36.1	97.8	74.3	100	20.1	68.7
B (72)	1.4	88.9	44.4	84.7	73.6	100	45.8	95.8
C (69)	2.9	87.0	68.1	92.8	88.4	100	66.7	91.3
D (64)	3.1	56.3	9.4	100	76.6	100	12.5	85.9
E (74)	9.5	52.7	29.7	94.6	71.6	100	31.1	71.6
F (54)	7.4	96.3	57.4	98.1	83.3	100	50	90.7

FD = fusidic acid, GAT = gatifloxacin, GEN = gentamicin, LZD = linezolid, MUP = mupirocin, Q/D = quinupristin/dalfopristin, SXT = sulfamethoxazole/trimethoprim, CHL = chloramphenicol.

The antibiotic susceptibilities of the isolates were categorized into patterns encompassing all the tested antimicrobials, table 3. The most common pattern observed was that coded 1 (109/512, 21.3%) followed by pattern 16 (100/512, 19.5%) then patterns 9 (54/512, 10.5%) and 4 (49/512, 9.6%). This table could serve to delineate the most probable pattern of the resistance per hospital thus aiding in choice of empirical therapy.

**Table 3:** Percent of the most common Antibiotic Susceptibility Patterns per hospital

Antibiotic Susceptibility Pattern	A (179)	B (72)	C (69)	D (64)	E (74)	F (54)
**1 **GAT/LZD/MUP/QD/CHL	22.3	33.3	15.9	29.7	4.1	24.5
**2 **GAT/LZD/QD	12.8	0	0	0	1.4	0
**4 **GAT/LZD/MUP/QD	12.8	4.2	4.3	9.4	16.2	4.1
**9 **LZD/MUP/QD/CHL	9.5	2.8	5.8	23.4	18.9	2.0
**11 **GAT/GEN/LZD/MUP/QD/C	11.2	0	1.5	0	0	8.2
**16 **GAT/GEN/LZD/MUP/QD/SXT/CHL	10.1	25	49.3	3.1	10.8	38.8

FD = fusidic acid, GAT = gatifloxacin, GEN = gentamicin, LZD = linezolid, MUP = mupirocin, Q/D = quinupristin/dalfopristin, SXT = sulfamethoxazole/trimethoprim, CHL = chloramphenicol. A-F represent the hospital codes

The emergence of antimicrobial resistance among a number of bacterial pathogens changes the way we practice medicine and places some of our patients at risk of dying from their infections. The overuse and misuse of antibiotics are major contributing factors to bacterial resistance; therefore it is incumbent on each of us to use antibiotics judiciously and appropriately. Judicious antibiotic use means that antibiotics are prescribed only when indicated and that the drug chosen is the most narrow spectrum agent that will be effective. Appropriate use means choosing not only the correct antibiotic but also the appropriate dose and duration, factors that can influence the development and carriage of resistant organisms[56,57]. These "resistotype" data could be complemented with "genotype" data and together, they could be used to exchange profiles across borders rather than actual material exchange.

**Figure 2:**
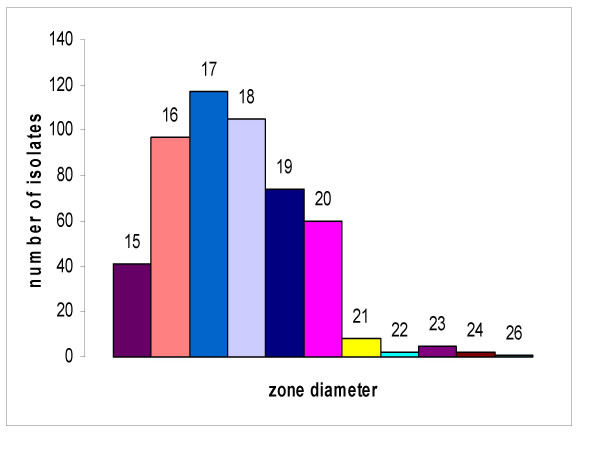
**Zone diameters of the isolates against vancomycin disc**. The numbers above the columns are the diameters of the zones.

**Figure 3:**
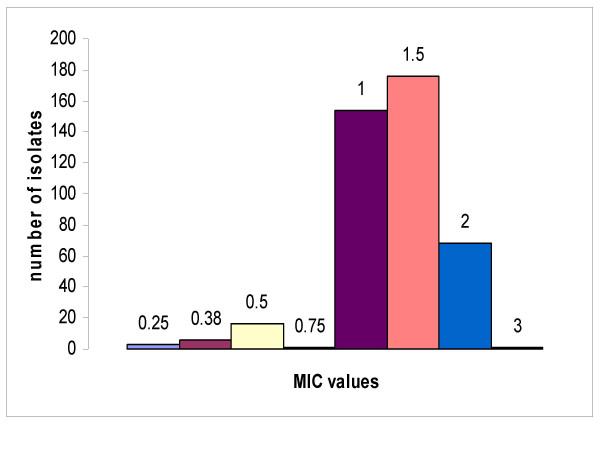
**MIC of isolates to vancomycin as determined by the E-test**. The numbers above the columns are the MICs in μg/mL.

The zone diameters of the isolates to the vancomycin discs were determined and are displayed in figure 2. Zone diameters ranged from 15 to 26 with most of the isolates giving zones ranging from 16 to 19 mm. This is in compliance with the CLSI standards for vancomycin (≤15 mm) indicating that none of the isolates was resistant to vancomycin. However, as the disc diffusion method would not differentiate strains with reduced susceptibility to vancomycin (MICs 4 to 8 μg/mL) from susceptible strains, the MIC was determined using the E-test to test for the presence of any isolate with decreased susceptibility to the antibiotic. The results of the vancomycin E-test for the isolates are shown in figure 3. The histogram shows that all isolates were susceptible to vancomycin with no evidence of reduced susceptibility to the drug. The MICs fell in the range of 0.25 to 3 μg/mL with most isolates in the 1 and 1.5 μg/mL groups.

**Figure 4:**
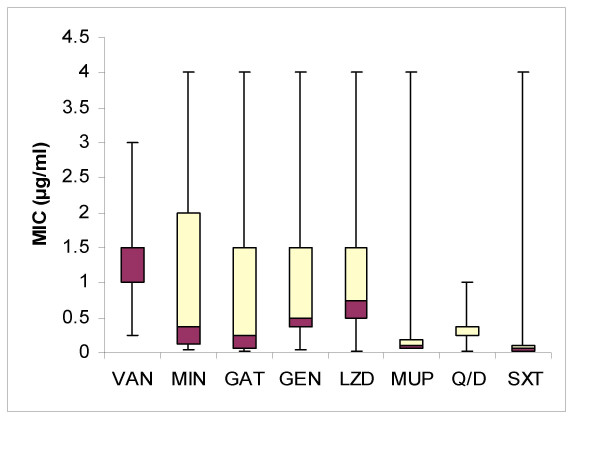
**MICs for the tested antibiotics**. VAN = vancomycin, MIN = minocycline, GAT = gatifloxacin, GEN = gentamicin, LZD = linezolid, MUP = mupirocin, Q/D = quinupristin/dalfopristin, SXT = sulfamethoxazole/trimethoprim.

The results of the E-test were as shown in figure 4 where the line in between the two coloured areas of each box represent the median or MIC50, the light area represents isolates having MIC at the range between 50^th ^percentile and 75^th ^percentile, while the dark area represents isolates having MIC at the range between 50^th ^percentile and 75^th ^percentile. Minocycline MIC ranged from 0.032 to 8 μg/mL, meaning that all were susceptible except 3 isolates which were intermediate (8 μg/mL). They showed 2 peaks, one at 0.094 – 0.125 μg/mL and the other at 2 – 3 μg/mL. Gatifloxacin MICs for susceptible strains ranged from 0.016 to 4 μg/mL indicating that some isolates are in the intermediate range (4 μg/mL). Most isolates had MICs in the range of 0.064 – 0.094 μg/mL and 1.5 μg/mL. Gentamicin MICs for susceptible isolates ranged from 0.047 to 4 μg/mL which are within the susceptible range by CLSI ≤ 4 μg/mL with most in the 0.035 to 0.5 μg/mL range. On the other hand MICs for linezolid disc diffusion susceptible isolates ranged from 0.016 to 4 μg/mL which is within the susceptible range according to CLSI standards (≤4 μg/mL) with most isolates falling in the 0.5 μg/mL group. As for mupirocin, MIC ranged from 0.064 to 6 μg/mL with only one isolate giving 6 μg/mL. As susceptibility breakpoints for mupirocin have not yet been established by CLSI, the following widely accepted breakpoints were used: ≤ 4 mg/l (susceptible), 8–128 mg/l (low-level resistance) and ≥ 256 mg/l (high-level resistance)[55]. Thus only one tested isolate showed decreased susceptibility not mounting to low-level resistance and most of the other isolates had MICs in the range of 0.064 to 0.094 μg/mL. Similarly, MIC for Quinupristin-dalfopristin ranged from 0.025 to 1 μg/mL which is also within the susceptible range (≤1 μg/mL) with most isolates in the 0.25–0.38 μg/mL range. Finally, trimethoprim/sulfamethoxazole MIC ranged from 0.012 to 0.4 μg/mL, which is also much lower than the CLSI standards for resistance (≥4/76 μg/mL). There was no evident preponderance of any MIC value.

In an attempt to study the antibiotic susceptibility trend over time, the study isolates were segregated into 4 groups according to the time of sample acquisition, each group covering a period of 6 months of collection time. The overall antibiotic susceptibility of each group to the tested antimicrobials was tabulated in table 4. From the table, it appears that the susceptibility to gatifloxacin markedly declined over the studied intervals especially the forth period (from 96.4% to 51.2%), this is not surprising, given the reported rapid acquisition of MRSA to resistance to fluoroquinolones. There was a trend towards declining susceptibility to linezolid also (from 98.2% to 92.7%). On the other hand, there was a trend towards increased susceptibility to gentamicin which was quite remarkable (14.5% to 46.3%) and a less evident one for sulfamethoxazole/trimethoprim (21.8% to 39.0%). These probably signify regaining some value of these antimicrobials with decreased usage.

**Table 4:** Trend over time of percent antibiotic susceptibility according to collection period

Isolation period (No.)	FD	GAT	GEN	LZD	MUP	Q/D	SXT	CHL
1 (55)	3.6	96.4	14.5	98.2	85.5	100	21.8	83.6
2 (206)	3.4	88.3	36.9	98.1	80.6	100	30.6	72.3
3 (210)	5.2	83.8	51.4	93.8	75.6	100	36.2	82.3
4 (41)	4.8	51.2	46.3	92.7	82.9	100	39.0	82.9

FD = fusidic acid, GAT = gatifloxacin, GEN = gentamicin, LZD = linezolid, MUP = mupirocin, Q/D = quinupristin/dalfopristin, SXT = sulfamethoxazole/trimethoprim, CHL = chloramphenicol.

Thus, the good news is that bacterial resistance is to some degree reversible. Reducing antibiotic use should be effective in combating resistance development, because resistant bacteria have no competitive advantage in the absence of antibiotic exposure and because colonization with resistant pathogens is usually transient. Because carriage of these resistant bacteria resolves spontaneously, susceptible strains eventually replace resistant strains in the absence of antibiotic exposure. Antibiotic restrictions do not always guarantee that antimicrobial resistance will disappear, however, as demonstrated by a report from the UK [58]. The reasons for this are not clear, although it may be because the determinants of some antibiotic resistance are genetically linked to other resistance determinants.

## Conclusion

None of the 512 tested isolates had reduced susceptibility to vancomycin with most MICs lying in the 1 – 1.5 range. Linezolid and quinupristin-dalfopristin are the most effective antibiotics tested against inpatient isolates while gatifloxacin and quinupristin-dalfopristin seem to be the most effective against outpatient isolates. Trends over time show a tendency towards decreased susceptibility to gatifloxacin and linezolid with increasing susceptibility to gentamicin and sulfamethoxazole/trimethoprim.

Quinupristin/dalfopristin and linezolid are two valuable additions to our antimicrobial armamentarium, but resistance has already been described. To preserve their value, their use should be limited to those rare cases where they are clearly needed.

Differences noted in the susceptibility of the isolates from different hospitals probably reflects the different patterns of antibiotic usage and thus development of resistance in these hospitals. Fusidic acid, the local antibiotic, gentamicin and trimethoprim/sulfamethoxazole should not be relied upon for treatment of MRSA infections, at least empirically as the percentage of susceptible isolates is very low. Approximately one forth of the isolates are no longer susceptible to mupirocin used for eradication of the carrier state reflecting resistance developing after widespread use. Keeping these resistotype data in mind while prescribing antibiotics for MRSA infected patients should aid in the prevention of its spread and abiding by the same principles kingdom-wide could limit its deleterious effects. An ongoing study by the same group is genotyping these MRSA isolates for delineating their genetic origins and perhaps their transmission dynamics as they constitute a precious resource for further investigations.

## Declaration of competing interests

The author(s) declare that they have no competing interests.

## Authors' contributions

MB designed the study, carried out the testing, performed the statistical analysis and interpretation of data and drafted the manuscript. MK participated in antibiotic testing and statistical analysis. AF conceived of the study, and participated in the preparation of the settings. All authors read and approved the final manuscript.
